# Prevalences of Pospiviroid Contamination in Large Seed Lots of Tomato and Capsicum, and Related Seed Testing Considerations

**DOI:** 10.3390/v11111034

**Published:** 2019-11-06

**Authors:** David Dall, Lindsay Penrose, Andrew Daly, Fiona Constable, Mark Gibbs

**Affiliations:** 1 Australian Government Department of Agriculture, GPO Box 858, Canberra ACT 2601, Australia; lindsay.penrose@agriculture.gov.au (L.P.); mark.gibbs@agriculture.gov.au (M.G.); 2 NSW Department of Primary Industries, Elizabeth Macarthur Agricultural Institute, Woodbridge Road, Menangle NSW 2568, Australia; andrew.daly@dpi.nsw.gov.au; 3 Agriculture Victoria Research, AgriBio, 5 Ring Road Bundoora VIC 3083, Australia; fiona.constable@ecodev.vic.gov.au

**Keywords:** Pospiviroid, seed-borne pathogens, biosecurity

## Abstract

Analyses of pospiviroids in commercial seed lots of tomato and capsicum, determined by testing of 12,000 to 40,000 seeds per lot, have enabled the development of empirically-derived distribution curves for the observed prevalences of viroids in those commodities. Those distribution curves can be considered in conjunction with statistically-based estimates of detection that would be achieved using other sample sizes. Statistical calculations using binomial distributions show that sample sizes of 3000 and 9400 seeds allow detection of viroid prevalences as low as 0.1% and 0.032%, respectively, with 95% confidence. Applying those calculations to observed viroid prevalences in contaminated tomato seed lots, it is estimated that the use of sample sizes of 3000 and 9400 seeds would detect 15% and 42%, respectively, of the contaminated seed lots identified using the larger sample sizes of approximately 20,000 seeds reported in this study. It is concluded that the higher costs associated with testing of larger sample sizes represent a worthwhile investment in agricultural biosecurity.

## 1. Introduction

Many viruses and viroids are transmitted vertically to seeds produced by infected plants, and through them to progeny plants [1,2]. Trade and transport of contaminated seeds can be of “considerable ecological significance” [2] with respect to the spread of those disease agents to new locations, and the initiation of outbreaks of disease [3]. As a consequence, many countries maintain import measures to reduce the risks of imported seed carrying viruses and viroids across their borders.

Consistent with this practice, Australia maintains requirements for testing of various imported vegetable seeds for bacterial and viral pathogens, including *Candidatus* Liberibacter solanacearum, *Cucumber green mottle mosaic virus* and *Melon necrotic spot virus*.

For tomato (*Solanum lycopersicum*) and capsicum (*Capsicum annuum* and related species) seeds, Australia requires testing for the presence of six pospiviroids, namely, *Columnea latent viroid* (CLVd), *Pepper chat fruit viroid* (PCFVd), *Potato spindle tuber viroid* (PSTVd), *Tomato apical stunt viroid* (TASVd), *Tomato chlorotic dwarf viroid* (TCDVd) and *Tomato planta macho viroid* (TPMVd). The testing regime can also result in detection of *Citrus exocortis viroid* (CEVd), which does not require regulatory exclusion in seed in Australia. A recent compilation of test results [4] showed that significant numbers of pospiviroid-contaminated tomato and capsicum seed lots were identified in the period 2012–2016 among seed lots proposed for import, reported details of recorded incidences (numbers of detections), and estimated prevalences (rates of contamination) of PSTVd.

This report describes an analysis of an extended dataset comprising the estimated prevalences of the five pospiviroid species detected by the same laboratories in Australia in large seed lots (≥60,000 seeds) of tomato and capsicum. It further uses these data to estimate efficiencies of detection for assurance strategies that are currently in place in Australia, and for others elsewhere proposed for use.

## 2. Materials and Methods

Samples from commercially-produced tomato and capsicum seed lots that were proposed for importation into Australia for planting were tested for the presence of pospiviroids. Details of the origins and characteristics of seed lots, and of sampling and testing processes, have been provided previously [4]. Briefly, 289 large tomato seed lots, each comprising at least 66,000 seeds, and 63 large seed lots of capsicum, each comprising at least 60,000 seeds, were tested. Reported data for tomato seed lots are based on tested samples of 13,200 to 20,000 seeds per lot (mean = 19,461), dependent on seed lot size, each tested as independent sub-samples of 400 seeds by conventional reverse-transcription PCR (RT-PCR), with positive results confirmed by sequencing of amplicons. Data for capsicum seeds are based on samples of 12,000 to 40,000 seeds (mean = 19,371) tested in the same manner. The viroid prevalence (fraction of contaminated seeds) within each contaminated seed lot was estimated by fitting test results to Markov Chain Monte Carlo models [4]. Data reported here represent tests of a cumulative total of more than 6.8 million seeds by the two participating laboratories.

## 3. Results and Discussion

### 3.1. Incidence and Prevalence of Pospiviroid Contamination

Forty nine of the large tomato seed lots (17.0%) were found to contain at least one of five detected pospiviroid species [4]. PSTVd was the most commonly found, being present in 25 (51%) of the contaminated seed lots; CEVd, TCDVd, PCFVd, and CLVd were recorded in 25%, 10%, 8%, and 6% of seed lots, respectively. Fourteen capsicum seed lots (22.2%) were found to be contaminated with PSTVd, PCFVd, or CLVd at incidences of 57%, 36%, and 7%, respectively, in the total of contaminated seed lots. TCDVd was not detected in capsicum seed lots, and TASVd and TPMVd were not detected in any of the seed lots considered in this report. However, TASVd was detected in smaller seed lots tested in the same Australian program [4].

The estimated prevalences of pospiviroid contamination (i.e., fractions of viroid-contaminated seeds in individual seed lots) ranged from maxima of 2.12 × 10^−3^ (0.212%) and 4.76 × 10^−3^ (0.476%) for tomato and capsicum seed lots, respectively, to the minimum estimable prevalence of 4 × 10^−5^ (0.004%; Supplementary Table S1).

**Figure 1:**
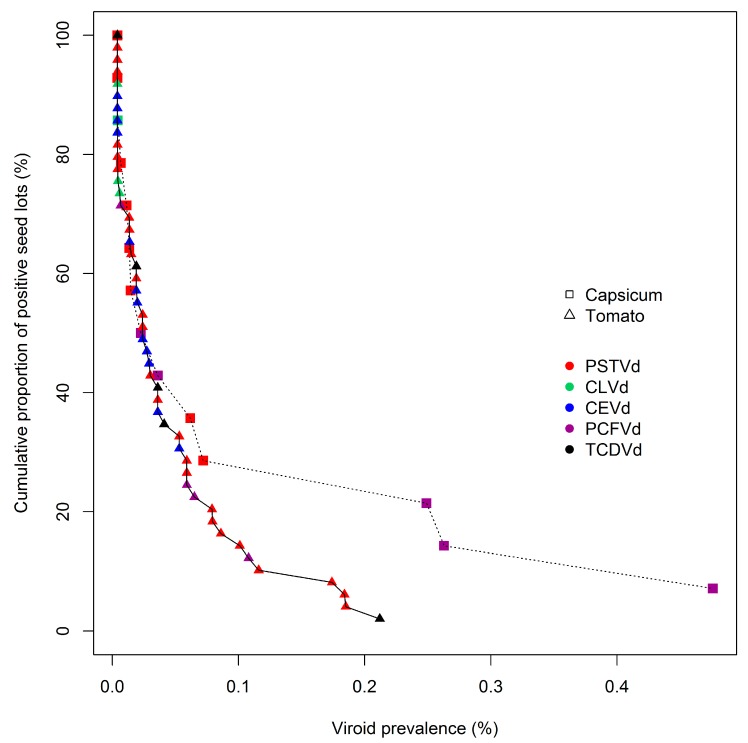
Cumulative distribution curves of viroid prevalences in contaminated tomato and capsicum seed lots presented for importation into Australia. Curves display the cumulative proportion by percentage (y-axis) of contaminated seed lots with viroid prevalences greater than, or equal to, the fractional rate (%) of prevalence identified on the x-axis. See text for full names of viroids.

The frequency distribution of viroid prevalence levels in individual seed lots was analyzed across the entire population of contaminated lots for each host species. To visualize those distributions, cumulative prevalence distribution curves were developed (Figure 1). The data points for the distributions were derived by ranking contaminated seed lots according to their estimated level of viroid prevalence (Supplementary Table S1), and then calculating their percentile rank on the basis of the percentage of all seed lots possessing the same or greater levels of contamination. Each data point was then plotted by prevalence of contamination (x-axis), and percentile rank (y-axis; Figure 1). The seed lots with the greatest levels of contamination are thus represented by points at the lower right of each curve, and seed lots with the smallest levels by points at the upper left. In summary, each curve in Figure 1 displays the percentile rank (y-axis) of contaminated tomato and capsicum seed lots (triangular and square markers, respectively) with viroid prevalences greater than, or equal to, a given fractional level (x-axis). Therefore, as shown, 100% of seed lots had a prevalence of contamination greater than, or equal to, 0.004%, while 20% of contaminated tomato seed lots had viroid prevalences of approximately 0.075% or above, and 20% of capsicum seed lots had prevalences of about 0.25% or above. The identities of the pospiviroid species depicted at each data point is denoted by marker color. The close alignment of these curves indicates that, within the limits of sample number availability, the two commodities apparently possessed very similar frequency distributions of viroid prevalence.

Overall, these data provide a comprehensive assessment of the incidence and prevalence levels of pospiviroid contamination in large commercial seed lots of tomato and capsicum seeds from sources of origin around the globe.

### 3.2. Assessment of Efficiencies of Different Sampling Regimes for Detection of Pospiviroids in Large Seed Lots

The availability of the cumulative prevalence distribution curves, provided in Figure 1, permits assessments to be made of the predicted efficiencies of different sampling regimes for detecting pospiviroids in the population of contaminated seed lots described here.

**Figure 2:**
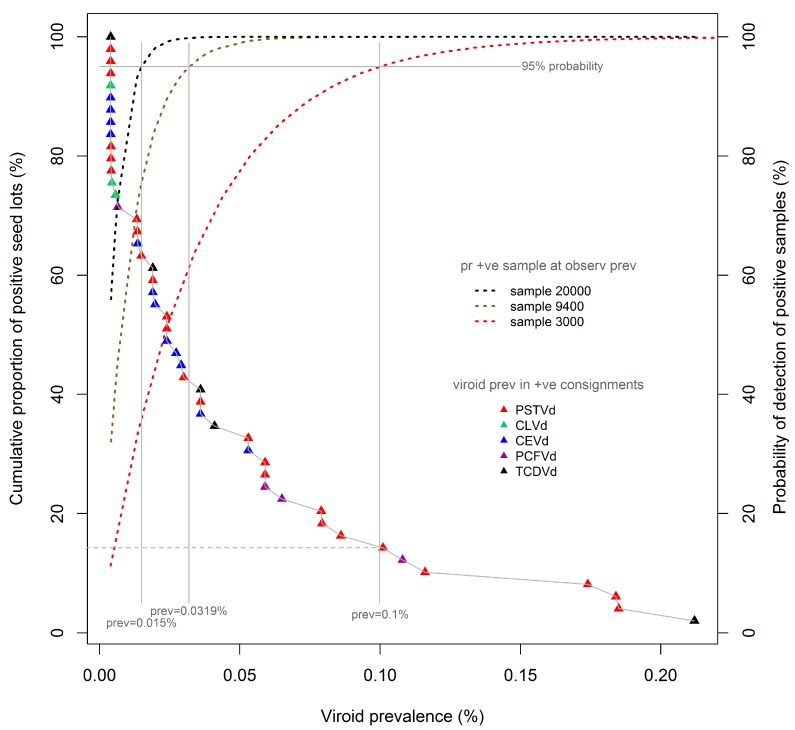
Cumulative distribution curve of viroid prevalence for contaminated tomato seed lots, overlaid with statistical confidence level (CL) curves (dotted lines) for three test sample sizes. See Figure 1 and text for interpretation.

Figure 2 reproduces the cumulative prevalence distribution of tomato seeds, shown in Figure 1, overlaid with detection probability curves (dotted lines) for three test sample sizes commonly adopted in international and Australian protocols, namely, 3000, 9400, and 20000 seeds. The probability curves were calculated using binomial distributions, which are applicable to the large seed lots under consideration here. Each probability curve is specific to the associated test sample size (e.g., 3000 seeds), and shows the probability (also known colloquially as the “level of confidence”; right vertical axis) of detecting the presence of viroids in seed lots contaminated at different prevalence rates (%, x-axis) when using that sample size. Thus, for each sample-specific probability curve, the prevalence of contamination that is detectable at a nominated level of confidence (e.g., 95%) can be determined by extending a vertical “plumb line” from the appropriate point of the CL curve to the x-axis. Using this methodology, it can be observed that a viroid prevalence of as low as 0.1% will be detected with 95% confidence using a test sample size of 3000 seeds. The same methodology indicates that viroid prevalences as low as 0.032% and 0.015% can be detected with the same level of confidence in samples sizes of 9400 and 20,000 seeds respectively.

The proportion of the laboratory-identified contaminated seed lots to which such prevalence levels would be expected to apply can be determined by extending a horizontal line (Figure 2, grey dashed) from the point of intersection of the “plumb line” and the cumulative distribution curve to the left vertical axis. Thus, the use of a sample size of 3000 seeds would be expected, at a confidence level of 95%, to have detected approximately 15% of the identified contaminated seed lots, namely, the seven lots with a viroid prevalence of 0.1% or more. The same rationale and method indicates that use of a sample size of 9400 seeds would be expected to have detected approximately 42% of all contaminated seed lots, in this instance being the 20 lots with an estimated viroid prevalence of 0.032% or more.

The discussion provided above has been based on a confidence level (probability of detection) of 95% (0.95) and a sample size of 3000 seeds, as (i) this sample size aligns with regimes established by some international agencies which countenance minimum acceptable test samples of this size [5,6], and (ii) it is more difficult to exemplify the concept diagrammatically at higher confidence levels because of the shallow gradients of confidence level curves at higher confidence levels.

Nevertheless, we note that for microbial pests with moderate to high potential for spread, and significant potential consequences for production systems and/or the environment, a confidence of detection of 99% (0.99) would appear to be a more appropriate regulatory setting. Applying this level of confidence of detection to the contaminated seed lot population described here would predict detection rates of 20% and 53% of contaminated seed lots using sample sizes of 9400 and 20,000 seeds respectively, being those lots with estimated contamination rates at or above 0.049% and 0.023%. At the same level of confidence, the use of a sample size of 3000 seeds would predict detection of 8% of contaminated seed lots, that is, the four seed lots with contamination rates above 0.15%.

Finally, knowledge of the viroid prevalence distribution can also be used to determine the average probabilities of detecting contaminated lots within selected “binned” proportions of the observed prevalence range. As shown in Table 1, the average probabilities of detecting individual seed lots within binned 33-percent quantiles of the population of contaminated tomato seed lots (Supplementary Table S1) are substantially affected by sample size. Thus, for the one-third of contaminated seed lots with highest viroid prevalences, the average probabilities of detecting contaminated lots are relatively similar at 0.909, 0.998 and 1.00 for sample sizes of 3000, 9400 and 20000 seeds respectively. In contrast, for the one-third of contaminated seed lots with lowest viroid prevalences, the average probabilities are highly divergent at 0.519, 0.746, and 0.862 for the same respective sample sizes.

Although the identified prevalences of viroid contamination of tomato and capsicum seed lots reported here are relatively low (but also broadly consistent with those recorded for *Cucumber green mottle mosaic virus* in large seed lots of cucurbits [7]), it is considered that their poorly regulated entry into Australia would pose a genuine risk of establishing foci of viroid infections in production settings.

The estimates of transmission rates of pospiviroids from contaminated seeds are highly variable. In a detailed study of characteristics of infection of tomato (cv “Beefsteak”) with PSTVd, Simmons et al. [8] reported high levels of transmission (62–69%) to seeds of deliberately infected plants, and resultant infection rates of 50.9% in seedlings from those seeds. These data indicate a PSTVd transmission rate of about 75% from contaminated seed to progeny seedlings.

Tolerable levels of pathogen inoculum threshold are recognized as being extremely difficult to quantify, with factors such as identities of host and pathogens, routes of transmission, and environmental and edaphic factors, potentially all having some influence [1]. Research on *Lettuce mosaic virus* in lettuce production systems in California [9] reported that seed stocks with contamination rates of 0.1% were unsuitable for use in systems with a long season of production, such that the use of seed lots with an indexed rate of zero infection in 30,000 germinated seedlings was adopted as a standard.

If a similar standard (that is, less than 1 infection per 30,000 seedlings; equivalent to <0.0033%) was adopted for pospiviroids in tomato, the data of Simmons et al. [9] suggest that tomato seed lots should have a prevalence of contamination no higher than about one in 22,500 seeds (0.0044%). Allowing a 75% transmission rate (see above), it could be anticipated that the resultant seedlings would then be infected at a rate of no more than 0.0033% (=1 in 30,000 plants). We note, however, that the transmission of viroids in production systems may be less efficient than that of LMV, which is disseminated by several aphid vectors; as such is it not unreasonable to accept somewhat higher levels of viroid presence in seeds.

The current Australian system of testing using a sample size of 20,000 seeds and the protocol described by Constable et al. [4] has been calculated to detect rates of contamination of 0.015% with 95% confidence and of 0.023% with 99% confidence, representing multiples of about 4- to 7-fold above the tolerances proposed for LMV; the regime can, however, detect contamination prevalence as low as 0.004%, although only 55% of consignments with viroid prevalence at this level would be expected to be detected using a test sample of 20,000 seeds.

Very large volumes of vegetable seeds are imported into Australia each year. Records of the Australian Government Department of Agriculture indicate that approximately 890 kilograms of tomato seed (approximately 265 million seeds) were imported in 2018. Assuming an overall prevalence of contamination of less than 0.005%, no more than about 13,000 viroid-contaminated seeds were likely to have gained entry into Australia in that year. As noted above, pospiviroid transmission through tomato and capsicum seeds is not fully effective, so a lesser number of infected seedlings is likely to have been produced, and given seedling attrition, the overall number of infected plants is likely to have been low. Reports of pospiviroid infections have been rare in Australia since the existing seed testing requirements were implemented, suggesting that the current test sample size is broadly appropriate.

The data and considerations provided here lead us to conclude that tests of larger sample sizes reveal instances of pospiviroid contamination that are unlikely to be detected with smaller samples, and therefore represent a worthwhile investment in agricultural biosecurity.

## Figures and Tables

**Table 1:** Effect of seed sample size on average probabilities of detecting contaminated tomato seed lots within ranked bins across the observed viroid prevalence distribution. Each bin comprises one-third of the total of contaminated tomato seed lots.

Binning of Contaminated Seed Lots	Seed Sample Size		
	3000	9400	20,000
Least-contaminated one-third of seed lots	0.519	0.746	0.862
Intermediately-contaminated one-third of seed lots	0.706	0.935	0.991
Most heavily contaminated one-third of seed lots	0.909	0.998	1.000

## Supplementary Materials

The following are available online at https://www.mdpi.com/1999-4915/11/11/1034/s1, Table S1: Ranked prevalence estimates for pospiviroids in contaminated tomato and capsicum seed lots.
