# Supplementary material for: Prevalences of Pospiviroid Contamination in Large Seed Lots of Tomato and Capsicum, and Related Seed Testing Considerations
Source: Viruses. 2019 Nov 6;11(11):1034. doi: 10.3390/v11111034 (PMC6893411; doi:10.3390/v11111034)
Supplement: Supplementary file 1 [file viruses-11-01034-s001.pdf]

Supplementary Table S1. Analyses of pospiviroid detections in tomato and capsicum seed lots

1.1 Analysis of pospiviroid detections in tomato seed lots

Ranking by prevalence	Viroid identity	Estimated prevalence (%)	Cumulative contamination percentile	# seed tested
1	TCDVd	0.21200	2.04	20000
2	PSTVd	0.18500	4.08	18800
3	PSTVd	0.18400	6.12	18000
4	PSTVd	0.17400	8.16	22000
5	PSTVd	0.11600	10.20	20000
6	PCFVd	0.10800	12.24	20000
7	PSTVd	0.10100	14.29	20000
8	PSTVd	0.08600	16.33	20000
9	PSTVd	0.07930	18.37	18400
10	PSTVd	0.07900	20.41	20000
11	PCFVd	0.06500	22.45	20000
12	PCFVd	0.05900	24.49	20000
13	PSTVd	0.05900	26.53	20000
14	PSTVd	0.05900	28.57	20000
15	CEVd	0.05300	30.61	20000
16	PSTVd	0.05300	32.65	20000
17	TCDVd	0.04100	34.69	20000
18	CEVd	0.03600	36.73	20000
19	PSTVd	0.03600	38.78	20000
20	TCDVd	0.03600	40.82	20000
21	PSTVd	0.03000	42.86	20000
22	CEVd	0.02910	44.90	17200
23	CEVd	0.02730	46.94	17600
24	CEVd	0.02400	48.98	20000
25	PSTVd	0.02400	51.02	20000
26	PSTVd	0.02400	53.06	20000
27	CEVd	0.01980	55.10	19200
28	CEVd	0.01900	57.14	20000
29	PSTVd	0.01900	59.18	20000
30	TCDVd	0.01900	61.22	20000
31	PSTVd	0.01490	63.27	18800
32	CEVd	0.01360	65.31	13200
33	PSTVd	0.01350	67.35	20000

34	PSTVd	0.01330	69.39	19600
35	PCFVd	0.00650	71.43	20000
36	CLVd	0.00571	73.47	14000
37	CLVd	0.00440	75.51	18400
38	PSTVd	0.00417	77.55	19200
39	PSTVd	0.00408	79.59	19600
40	PSTVd	0.00408	81.63	19600
41	CEVd	0.00400	83.67	20000
42	CEVd	0.00400	85.71	20000
43	CEVd	0.00400	87.76	20000
44	CEVd	0.00400	89.80	20000
45	CLVd	0.00400	91.84	20000
46	PSTVd	0.00400	93.88	20000
47	PSTVd	0.00400	95.92	20000
48	PSTVd	0.00400	97.96	20000
49	TCDVd	0.00400	100.00	20000

1.2 Analysis of pospiviroid detections in capsicum seed lots

Ranking by prevalence	Viroid identity	Estimated prevalence (%)	Cumulative contamination percentile	# seed tested
1	PCFVd	0.47600	7.14	20000
2	PCFVd	0.26300	14.29	20000
3	PCFVd	0.24900	21.43	20000
4	PSTVd	0.07370	28.57	20000
5	PSTVd	0.06170	35.71	12000
6	PCFVd	0.03600	42.86	20000
7	PCFVd	0.02260	50.00	12400
8	PSTVd	0.01450	57.14	40000
9	PSTVd	0.01350	64.29	20000
10	PSTVd	0.01120	71.43	16000
11	PSTVd	0.00667	78.57	12000
12	CLVd	0.00426	85.71	18800
13	PSTVd	0.00400	92.86	20000
14	PSTVd	0.00400	100.00	20000
